# Diagnostic challenges in malaria detection: A comparative diagnostic performance of HRP2-based rapid diagnostic tests, microscopy, and PCR at Bichena primary hospital, Northwest Ethiopia

**DOI:** 10.1016/j.parepi.2026.e00485

**Published:** 2026-02-19

**Authors:** Awoke Minwuyelet, Delenasaw Yewhalaw, Getnet Atenafu

**Affiliations:** a Department of Biology, College of Natural and Computational Science, Debre Markos University, Debre Markos, Amhara, Ethiopia; b Tropical and Infectious Diseases Research Center, Jimma University, Jimma, Ethiopia; c School of Medical Laboratory Sciences, Faculty of Health Sciences, Jimma University, Jimma, Ethiopia

**Keywords:** Malaria, Diagnostic challenge, *P.Falciparum*, *P.Vivax*, RDT, Microscopy, PCR

## Abstract

**Background:**

Accurate malaria diagnosis is crucial for effective case management, strong surveillance, and progress toward elimination. However, in highland regions, diagnostic tools are underutilized or yield suboptimal performance. While hematological alterations are frequently observed in malaria, their role remains largely supportive rather than diagnostic. This study aimed to evaluate diagnostic challenges by comparing the performance of HRP2-based rapid diagnostic tests, microscopy, and PCR at Bichena Primary Hospital, Northwest Ethiopia, with hematological profiles examined as supportive indicators to help contextualize diagnostic performance.

**Materials and methods:**

A facility-based cross-sectional study was conducted between 31/12/2024 to 28/02/2025, with 274 participants enrolled through consecutive sampling. Socio-demographic data were collected using semi-structured questionnaires. The diagnostic evaluation used nested polymerase chain reaction (PCR) (from dried blood spots), microscopy (capillary and venous blood), histidine rich protein 2 (HRP2)-based rapid diagnostic tests (RDTs), and hematological profiling. Data analysis was carried out with Statistical Package for the Social Sciences (SPSS) version 25.0, assessing diagnostic accuracy through sensitivity, specificity, positive predictive value (PPV), and negative predictive value (NPV), while inter-test agreement was measured using Cohen's Kappa coefficient. Results were summarized in text, figures, and tables.

**Results:**

Higher prevalence of *Plasmodium* infections was detected in 23.4% of participants by PCR, 20.1% by microscopy and 19% by HRP2-antigen-based RDT. The HRP2-antigen based RDT showed lower sensitivity (79.1%), NPV (94.1%), and test accuracy (94.9%) compared to PCR. Similarly, microscopy exhibited high specificity and PPV (100%); however, the sensitivity was 85.9%, indicating that some true positives are missed compared to PCR. Moderate test agreement was observed between PCR and microscopy (κ = 0.904; *P* = 0.00) but weak agreement between PCR and RDTs (κ = 0.847). Hematological analysis revealed a significantly lower platelet count among PCR-confirmed malaria cases (*P* < 0.05), suggesting a supportive association rather than diagnostic utility.

**Conclusions:**

Both HRP2-antigen based RDTs and microscopy demonstrated lower sensitivity compared to PCR. RDTs showed the lowest diagnostic potential for *P. falciparum*, mixed and even *P. vivax* infections, this may be due to low parasitemia and possible pfhrp2 deletions. Hematological parameters, particularly platelet count, may serve as complementary indicators to support clinical suspicion but should not replace parasitological or molecular diagnosis. Further investigation of pfhrp2/pfhrp3 deletions is critical to inform the selection of appropriate diagnostic tools in the area.

## 1 Introduction

Malaria continues to pose a major public health challenge worldwide despite sustained control efforts over recent decades. The World Malaria Report 2025 estimates that approximately 282 million malaria cases and about 610,000 deaths occurred globally in 2024, reflecting an increase malaria burden compared with the previous year, which has been attributed to a combination of climate variability, health system disruptions, insecticide resistance, and diagnostic challenges in endemic regions. Despite large-scale deployment of vector control interventions and rapid diagnostic tests, the African Region continues to account for nearly 94–95% of global malaria cases and deaths with children under five years of age remaining the most affected population (WHO, 2025).

In Ethiopia, malaria remains endemic across large parts of the country. Approximately 75% of the landmass is classified as malaria-endemic, placing nearly 69% of the population at risk of infection. Between 1 January and 20 October 2024, more than 7.3 million malaria cases and 1157 deaths were reported nationwide (case fatality rate: 0.02%), representing one of the highest annual case counts recorded in recent years (WHO, 2025; WHO, 2024a).

The malaria epidemiology in Ethiopia is characterized by a dynamic interplay between *Plasmodium* species, with *P. falciparum* and *P. vivax* being the most prevalent. Recent evidence highlights a complex and heterogeneous transmission landscape, marked by substantial regional variation in species distribution and prevalence trends. Although Ethiopia experienced a marked decline in malaria cases between 2017/18 and 2021/22, a resurgence was observed in 2022/23 (Mandefro et al., 2023; Minwuyelet et al., 2025). Historically, *P. falciparum* has been the dominant species nationwide; however, *P. vivax* is increasingly reported in several regions, particularly in highland and fringe transmission areas. (Minwuyelet et al., 2025; Abere et al., 2025; Ketema et al., 2021).

Efforts to reduce the global malaria burden have made significant strides; however, challenges such as inaccurate and delayed diagnosis persist, alongside insecticide and antimalarial drug resistance (Sallam et al., 2025; Obeagu and Obeagu, 2024). These diagnostic inaccuracies continue to hinder malaria elimination efforts, particularly in low-income countries (Sallam et al., 2025; Yin et al., 2022; Sahira and Al-Abboodi, 2024; Han and Han, 2024). As a result, malaria remains a public health problem, and in 2024, over 247 million cases and 567,000 deaths were reported globally, primarily affecting children in Africa (WHO, 2024b).

Detecting malaria in regions with low endemicity poses significant challenges due to low parasite densities and a high prevalence of asymptomatic infections. Conventional diagnostic methods, such as microscopy and RDT, often fail to identify these low-density infections, which can contribute to ongoing transmission (Baptista et al., 2021; Foko et al., 2022; Mooney et al., 2022). This highlights the need for innovative solutions that improve accuracy, sensitivity, and speed in malaria diagnostics.

Recent advancements in diagnostic technology have enhanced the sensitivity, specificity, and overall efficiency of malaria detection using hematological biomarkers (Krampa et al., 2020; Mulatie et al., 2024). Biomarkers such as HRP2, plasmodial lactate dehydrogenase (pLDH), hemozoin, aldolase, and glutamate dehydrogenase (GDH) have gained attention for their potential to detect *Plasmodium* infections (Krampa et al., 2020; Harmonis et al., 2025; Mwesigwa et al., 2019). Simultaneously, molecular methods such as PCR provide highly accurate diagnostic alternatives because they can detect low levels of parasitemia (Fitri et al., 2022). However, despite their superior performance, PCR and other molecular tools are often impractical in resource-limited settings due to the necessity for specialized equipment and technical expertise (Chauhan et al., 2024).

Microscopy, the traditional gold standard, remains reliable for parasite detection but is time-consuming and requires skilled personnel (Han and Han, 2024; Chauhan et al., 2024). Among RDTs, the most commonly targeted antigens are PfHRP2, pLDH, and plasmodial aldolase. However, the diagnostic efficiency of these RDTs for malaria varies significantly across different geographical settings (Mwesigwa et al., 2019; Mouatcho and Goldring, 2013; Yimam et al., 2022; Ojurongbe et al., 2025; Slater et al., 2022; Ding et al., 2023; Opoku Afriyie et al., 2023). This may negatively impact malaria control and the commitment to eliminate the disease by 2030 (Woldesenbet et al., 2025; Organization WH, 2024; Oyegoke et al., 2022).

To address these challenges, it is essential to provide highly sensitive diagnostic tools suitable for resource-limited settings, to engage in cross-border collaboration, to enhance surveillance using existing diagnostic tools, and to develop alternative biomarkers to overcome malaria diagnostic challenges across diverse regions (Oyegoke et al., 2022; Wangdi et al., 2015).

Given the resurgence of malaria, evolving species distribution, and growing concerns regarding the challenge of HRP2-based RDTs, systematic evaluation of malaria diagnostic tools in routine healthcare settings in Ethiopia is needed…Therefore, the aim of this study was to assess the diagnostic challenges of malaria using different diagnostic tools in highland areas among patients attending Bichena Primary Hospital in the Amhara region of Northwest Ethiopia.

## 2 Materials and methods

### 2.1 Study area

The study was conducted at Bichena Primary Hospital, located in the town of Bichena, within the Enemay district. The area is located at an elevation of 2541 m above sea level (MAPCARTA, 2026). It serves four districts (Enemay, Debay Tilatgin, Enarg Enawuga, Shebele Berenta) and (Bichena town) administration with a population that exceeds the national standard of over 100,000 people. Malaria prevalence in the area fluctuated from year to year (Minwuyelet et al., 2025). Microscopy is the primary diagnostic tool for malaria in hospitals and health centers within the catchment area, while HRP2 antigen-based RDTs are used at health posts.

In this study, diagnostic tools such as microscopy, HRP2-antigen-based RDT, and PCR were used to evaluate the diagnostic challenges of malaria in the catchment area. This RDT is the main tool for diagnosing malaria, particularly for identifying *P. falciparum* through its HRP2 marker (Poti et al., 2020) and for detecting all *Plasmodium* species using pLDH.

### 2.2 Study design and period

A facility-based cross-sectional prospective study was conducted at Bichena Primary Hospital from 31 December 2024, to 28 February 2025.

### 2.3 Participant enrollment

The study included people of all ages and gender living within the hospital's catchment area. The source population comprised all study participants seeking medical care at Bichena Primary Hospital. The study population specifically consisted of those who present to the hospital's laboratory department with a request for a blood film examination.

### 2.4 Inclusion and exclusion criteria

The study population comprised individuals who visited the laboratory for malaria blood film examinations. Those who provided informed consent, either personally or through their legal guardians, were included in the study and asked to give blood for malaria diagnosis using light microscopy,PCR, HRP2-antigen-based RDTs, and hematological profiling. However, individuals who had taken antimalarial drugs in the month prior to the study period were excluded.

### 2.5 Sample size and sampling techniques/ sampling procedures

The method for comparing the sensitivity or specificity of two diagnostic tests at a 95% confidence level and 80% power was used to determine the sample size needed to assess the diagnostic performance of HRP2-antigen-based RDTs, microscopy, and nPCR (Hajian-Tilaki, 2014). Based on a prior study in Northwest Ethiopia, we assumed P₁ = 0.76, P₂ = 0.86, and Pₐ = 0.81, with Zα/2 = 1.96 and Zβ = 0.84, which reported PfHRP2/pLDH antigen based RDT sensitivity and specificity of 75.8% and 93.2% compared to PCR (Zeleke et al., 2023).

$$n={\left(\frac{\mathrm{Z\alpha}/2x\sqrt{2\mathrm{pa}\left(1-\mathrm{pa}\right)}}{{\left(p1-p2\right)}^{2}}+\frac{\mathrm{Z\beta}\sqrt{p1\left(1-p1\right)+p2\left(1-p2\right)\;}}{{\left(p1-p2\right)}^{2}}\right)}^{2}$$

The calculated sample size was *n* = 249; after adjusting for a 10% non-response rate, the final sample size was 274 individuals.

Patients who visited Bichena primary hospital and were suspected of having malaria were consecutively recruited after arriving with a lab request for malaria diagnosis in the laboratory department. Those who met the inclusion criteria were enrolled until the required sample size was achieved.

### 2.6 Operational definitions

The diagnostic challenges of malaria using HRP2-based RDTs refer to the limitations encountered when relying on HRP2 as a biomarker for detecting *Plasmodium* infection. These limitations may be due to factors such as HRP2 gene deletions, antigen persistence after treatment, and cross-reactivity, all of which can affect test accuracy (Krampa et al., 2020; Martiáñez-Vendrell et al., 2022).

Sensitivity refers to the test's ability to correctly identify those with the disease (true positive rate), while specificity refers to its ability to correctly identify those without the disease (true negative rate) (Ranadive et al., 2017; Unwin et al., 2020a). Positive predictive value (PPV) indicates the likelihood that someone who tests positive actually has the disease, whereas NPV indicates the likelihood that someone who tests negative is truly disease-free (Diallo et al., 2017a).

### 2.7 Data collection procedures

#### 2.7.1 Socio-demographic characteristics and other associated factors

Trained laboratory personnel were administered a pre-tested semi-structured questionnaire to gather socio-demographic and malaria-related data under the supervision of the principal investigator. The questionnaire was originally in English, translated into Amharic, and back-translated for analysis. Collected variables included sex, age, marital status, educational level (or that of parents for children under five), and household size. Additionally,the questionare include information on prevention methods (insecticide-treated bed nets (ITNs) ownership and use, indoor residual spray (IRS) history), previous malaria episodes, treatment, and relapse history. The questionnaire adapted with minor modifications from a previous study in Northeast Ethiopia (Minwuyelet et al., 2024). The participant's axillary temperature was also measured using a thermometer.

#### 2.7.2 Blood sample collection and processing

A 50 μL capillary blood sample was aseptically collected from each participant to prepare both thick and thin blood films on pre-labeled microscope slides for the detection and quantification of *Plasmodium* parasites. Additionally, 2 ml of venous blood were collected from each participant using EDTA test tubes for diagnosing malaria using HRP2-antigen based RDT, hematological profiling, and to prepare DBS for PCR assay. The DBSs were prepared by applying two separate drops of blood (50 μl each) into Ahlstrom TFN (Lasec®International (Pty) Ltd) filter paper for one patient (resulting in four spots for two patients on a five-circle filter paper, leaving one space free in between to avoid contamination) and allowed to air dry overnight. After dry, each DBS card was then individually sealed in a plastic bag with desiccant and stored in a refrigerator at −20 °C until it was transported to the molecular laboratory at TIDRC Sokoru, Jimma University, Jimma, Ethiopia.

#### 2.7.3 Laboratory analysis

##### 2.7.3.1 Malaria rapid diagnostic test and microscopy examinations

Malaria was diagnosed using Pf/Pv (HRP2/pLDH) Ag Combo RDT kits (CareStart™) by aseptically collecting 5 μl of blood, placing it in the sample well, and adding two drops of buffer. The tests were conducted and interpreted according to the manufacturer's instructions (WHO, 2015). This RDT is a rapid, three-band lateral-flow immunochromatographic test. The negative results were read after 20 min.

Malaria diagnosis was conducted using light microscopy (OLYMPUS CX22) by preparing thick (6 μl) and thin (2 μl) blood films on a single glass slide for each participant, which were allowed to air dry. The thin blood films were fixed with absolute methanol (99%), and both thick and thin films were stained with 10% fresh Giemsa solution for 10 min. Finally, the dried blood films were examined by the magnification of 100× using a light microscope to detect and identify *Plasmodium* species. After identifying the *Plasmodium* species at their developmental stage, the parasite density was also calculated. Gametocyte and asexual parasite densities were calculated against 500 and 200 leukocytes, respectively, assuming a standard mean white blood cell count of 8000 leukocytes per μl of blood (McKenzie et al., 2005), and converting the total number into parasites/μl (p/μl). A slide was considered negative if no *Plasmodium* parasites were seen after examining 200 fields. Blood smears were verified by at least two laboratory professionals before the results were reported to ensure the validity of the results. The discordant results were re-examined by a third senior medical laboratory technologist who was blinded to the initial result, and this result was considered final. The parasite density was calculated using the formula:

$$\text{Parasites}/\mathrm{\mu l}\;\text{of blood}=\frac{\text{Number of parasite counted}\times 8000\;\text{white blood cells}/\mathrm{\mu l}}{\text{Number of white blood cells counted}}$$

Gametocyte density per 1000 WBCs was calculated using the following formula: $\text{Gametocytes}\;\mathrm{per}\;1000\;\text{WBCs}=\frac{\text{Number of Gametocytes Counted}\times 1000}{\text{Number of WBCs Counted}}$

##### 2.7.3.2 Malaria nested PCR assay from DBS samples

Malaria diagnosis was also conducted using nested PCR. Genomic DNA was extracted for molecular analysis using the Chelex-100 method, as previously described by Wooden et al. (Wooden et al., 1993), after a few modifications. A 3 mm DBS sample was punched and was placed into pre-labeled 1.5 mL Eppendorf tubes. Then each sample was incubated overnight in 10% saponin (50 μL) and PBS (950 μL) at 4 °C, and rewashed with PBS (1000 μL), and air-dried. The pellet was treated with 20% Chelex® (150 μL) and DNA free water (100 μL), and then incubated at 95 °C for 10 min with intermittent vortexing. After centrifugation, 100 μL DNA in the supernatant was transferred to a labeled 1.5 mL Eppendorf tube and stored at −20 °C for nPCR analysis (Supplementary 1).

##### 2.7.3.3 DNA amplification and visualization

DNA amplification was performed using the TC9639 thermal cycler (Benchmark Scientific, Sayreville, NJ, USA) (Chua et al., 2016). To achieve this, the ToughMix® master mix was used, which contains optimized MgCl₂, dNTPs, hot-start polymerase, and stabilizers (Kamau et al., 2015). For genus-level detection, a 25 μL reaction included 2 μL DNA, 0.4 μM rPLU5/rPLU6 primers, 6 μL ToughMix®, and nuclease-free water were used. Cycling conditions were: 95 °C for 10 min, 35 cycles of 95 °C for 60 s, 58 °C for 60 s, 72 °C for 90 s, followed by 72 °C for 10 min, then 10 °C hold. Negative and positive controls were included in each run to check for contamination (Bharti et al., 2007).

Species-specific amplification was also performed using primers (rFAL1/2, rVIV1/2, rOVA1/2) to identify *P. falciparum, P. vivax*, and *P. ovale* (Rosanas-Urgell et al., 2010; Veron et al., 2009). The primers and their sequence were used according to Snounou et al. (Snounou and Singh, 2002) (Table 1). A 25 μL reaction, containing of 6 μL of PerfeCTa® qPCR ToughMix® (Quantabio), 0.4 μM of each primer, 2 μL of template DNA, and 14.6 μL of nuclease free water were also used. Thermal cycling was the same as genus-level PCR conditions but reduced to 30 cycles.

**Table 1:** Genus and species-specific primers sequences.

Name of species	Sequences (5′-3′)	Band size	References
rPLU5	CCT GTT GTT GCC TTA AAC TTC	1.5–1.6 kb (1.2kbp)	Snounou, G. et al. 2002 (Snounou and Singh, 2002)
rPLU6	TTA AAA TTG TTG CAG TTA AAA CG		
*P. falciparum*: rFAL1	TTA AAC TGG TTT GGG AAA ACC AAA TAT ATT	206 bp	
*P.falciparum*: rFAL2	ACA CAA TGA ACT CAA TCA TGA CTA CCC GTC		
*P.vivax*:rVIV1	CGC TTC TAG CTT AAT CCA CAT AAC TGA TAC	121 bp	
*P.vivax*: rVIV2	ACT TCC AAG CCG AAG CAA AGA AAG TCC TTA		
*P.ovale*:rOVA1	ATC TCT TTT GCT ATT TTT TAG TAT TGG AGA	780-800 bp	
*P oval*:rOVA2(rPLU2)	ATC TAA GAA TTT CAC CTC TGA CAT CTG		

Bp: base pair.

**Fig. 1:**
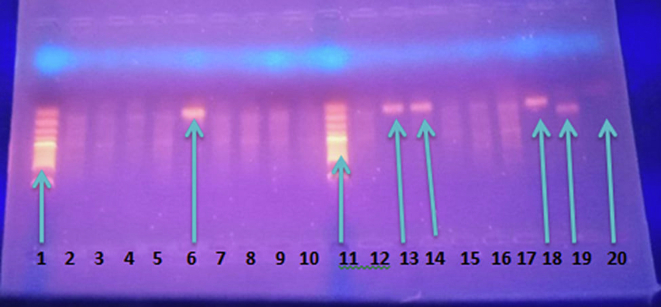
Nested PCR gel-electrophoresis *Plasmodium* species amplification result. This gel electrophoresis image showed nPCR amplification products for the detection of *Plasmodium vivax* and *Plasmodium falciparum* using species-specific primers. Lanes 1 and 11 (M): 100 bp DNA ladder used as a molecular size reference. Lanes 6, 13, 14, and 18: Positive bands at approximately 121 bp, indicating the presence of *P. vivax* DNA. Lane 19: Positive control for *P. falciparum*, showing a distinct band at ∼205 bp. Lanes 2–5, 7–10, 12, 15–17: No visible bands, indicating negative nPCR results or undetectable parasite DNA. Lane 20: Negative control showing no amplification, confirming absence of contamination.

Finally, PCR products were read on 1.5% agarose gel prepared with ethidium bromide in TAE buffer. After electrophoresis at 90 V for 75 min, DNA bands were visualized under UV and compared with a 100 bp ladder for species identification (Fig. 1).

In addition to HRP2-RDT, microscopy, and PCR, hematological profiling was performed as a complementary assessment in patients suspected of malaria. A Mindray BC-30s Auto Hematology Analyzer was used to evaluate hematological parameters. Hematological abnormalities were classified according to WHO criteria, with minimal adjustments for altitude (WHO, 2009).

### 2.8 Data analysis procedure

The data were coded, entered, cleaned, and analyzed using SPSS version 25 software (IBMCorp Ibm S, 2017). Descriptive statistics were used to summarize the distribution of both dependent and independent variables. The diagnostic performance of the diagnostic tools was assessed by calculating sensitivity, specificity, PPV, NPV and overall accuracy of RDT and microscopy using PCR as references. Additionally, inter-rater agreement between diagnostic tests was measured using Cohen's Kappa coefficient. Pearson correlation was applied to employ the correlation of hematological profiling and *Plasmodium* infection. Bivariate logistic regression was applied to explore associations between *Plasmodium* infection and other potential covariates. Frequency distribution was used to determine the prevalence of symptomatic *Plasmodium* infection. Finally, the finding was presented in texts, figures and tables. A *P*-value less than (0.05) was considered as statistically significant.

### 2.9 Data quality management

Questionnaire data quality control was assessed by conducting a pre-test before the data collection period. Training was given for the team before data collection. Before data entry, the returned questionnaire was checked for completeness and corrective measures were taken. All the test procedures and the interpretation of results were accomplished using standard operating procedures (SOP). The expiry date of reagents, materials and CareStart™ Malaria HRP2/pLDH (Pf/Pv) Combo (RDT) was checked daily before data collection takes place. The quality of Giemsa solution was assessed by using positive and negative control of blood films. All positive and negative microscope slides were reexamined by another Laboratory professional before the results were reported to avoid false results. nPCR assay was performed based on protocol and with negative and positive control.

### 2.10 Ethics approval and consent to participate

Ethical clearance for the study was obtained from the Institutional Review Board (IRB) of Debre Markos University, Research and Technology Transfer Directorate, on 30 December 2024 (Ref. No:DMU/RTTD/75/10/24; Protocol No: DMU/002/2017) (Supplementary 2). Verbal informed consent was obtained from all participants prior to enrollment, in accordance with the approved ethical guidelines.

## 3 Result

### 3.1 Characteristics of study participants

Among the total 396 malaria suspected patients, 274 met the inclusion criteria and consented to participate in the study. Of these, the majority (55.5%) of the study participants were males. Their age ranged from 7 months to 96 years and median age of study participants were 28.5 years (±19.5). Geographically, the majority (56.2%) resided in rural areas, and 35.8% were illiterate and the majority of (55.5%) were married. Household demographics revealed that the majority of participants (79.9%) lived in households with five or fewer family members. Majority 65.3% (*n* = 179) of did not own ITNs. A history of malaria contacted either personally or within the family was reported by 33.2% (*n* = 91). In terms of relapse, 21.9% (*n* = 60) had experienced recurrent malaria episodes, while 11.3% (*n* = 31) reported no relapse. Regarding clinical status at presentation, 41.2% (*n* = 113) were febrile (Table 2**)**.

**Table 2:** Study participants characteristics and malaria prevalence according to diagnostic tools.

Participants characteristics		Total (%)	*Plasmodium* detection rate per diagnosti tools		
			RDT (%)	Microscopy (%)	PCR (%)
Sex	Male	152 (55.5)	35(23)	38(25)	43(28.3)
	Female	122(44.5)	17(13.9)	17(13.9)	21(17.2)
Age Catagoty	<5	30(10.9)	7(23.3)	9(30)	11(35.7)
	5–14	23(8.4)	4(17.4)	4(17.4)	4(17.4)
	>15	221(80.7)	41(18.6)	42(19.1)	49(22.2)
Marital status	Single	54(19.6)	14(25.9)	13(24.1)	14(25.9)
	Married	152(55.5)	25(16.4)	27(9.8)	33(21.7)
	Windowed/divorced	18(6.6)	4(22.2)	4(22.2)	4(22.2)
Education status (Family for child)	Illiterates	98(35.8)	18(18.4)	21(21.4)	23(23.5)
	Read and write	61(22.3	9(14.7)	11(18)	13(21.3)
	Below high school	60(21.9)	14(23.3)	13(21.7)	15(25)
	graduated	55(20)	11(20)	10(18.2)	13(23.6)
Residence	Urban	119(43.4)	8(6.7)	11(9.2)	14(11.8)
	Rural	155(56.6)	44(28.4)	44(28.4)	50(32.3)
House hold size	<5	219(79.9)	33(15.1)	36(16.4)	42(16.9)
	>5	55(20.1)	19(34.5)	19(34.5)	22(40)
Ownerships of ITNs	No	179(65.3)	37(20.7)	38(21.2)	42(23.5)
	Yes	95(34.7)	15(15.8)	17(17.9)	22(23.2)
Utilization of ITNs	Daily	87(31.8)	14(16.1)	16(18.4)	19(21.8)
	Somtimes	8(2.9)	1(12.5)	1(12.5)	3(37.5)
Ever contacted (Even family)with malaria	No	183(66.8)	16(8.70	16(8.7)	25(13.7)
	Yes	91(33.2)	36(39.5)	39(42.8)	39(42.8)
Relaps	no	31(11.3)	4(12.9)	5(16.1)	5(16.1)
	Yes	60(21.9)	32(53.3)	34(56.7)	34(56.7)
Temprature	Non-febrile	161(58.8)	15(9.3)	15(9.3)	20(12.4)
	Febrile	113(41.2)	37(32.7)	40(35.9)	44(39.9)

Across all diagnostic tools, malaria prevalence was higher among males. Similarly, the majority of malaria cases were observed in children under five years of age, participants from rural areas, households with more than five members, and those with a history of malaria contact (Table 2).

### 3.2 Malaria prevalence across different diagnostic methods

**Fig. 2:**
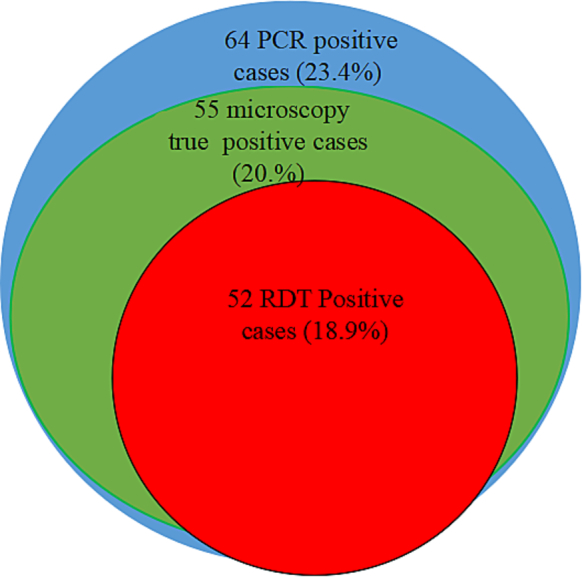
Prevalence of malaria using different diagnostic tools. Among 274 suspected patients tested using PCR, microscopy, and RDT (with PCR as the reference standard), microscopy missed 9 cases (false negatives). For RDT, of 52 positive cases reported, 49 were true positives, and there were 13 false negatives and 1 cases were also false positive.

The prevalence of malaria detected by PCR was 23.4% (95% CI, 18.4%–28.4%), compared to 20.1% (95% CI, 15.3%–24.8%) by microscopy and 18.9% (95% CI, 14.3%–23.6%) by HRP2-based RDT. Among the 64 malaria cases identified by nPCR, 76.6% (49/64) were also detected by both microscopy and HRP2-antigen based RDT. Additionally, 85.9% (55/64) of cases were detected by both PCR and microscopy, 79.8% (51/64) by nPCR and RDT, and 76.6% (49/64) by microscopy and HRP2-antigen based RDT (Fig. 2).

### 3.3 *Plasmodium* species detection rate using different diagnostic tools

**Fig. 3:**
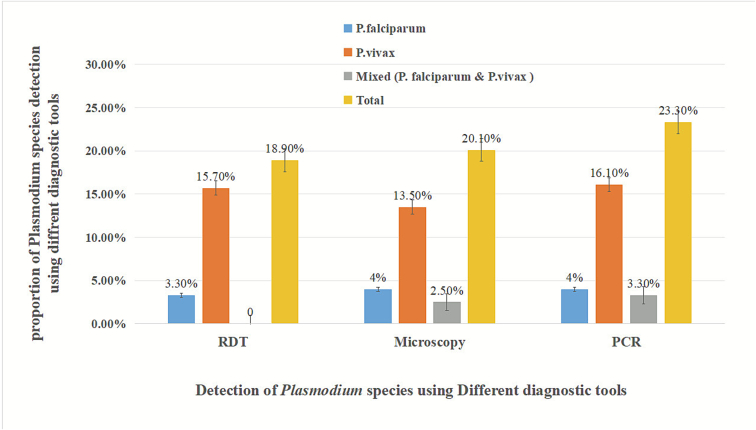
Distribution of *Plasmodium* infection by diagnostic methods.

At the species level, PCR identified *P. vivax* as the predominant species, accounting for 16.1% (95% CI: 11.7%, 20.4%). *P. falciparum* constituted 4.0% (95% CI: 1.7%–6.3%), and mixed infections (*P. falciparum* and *P. vivax*) also accounted for 3.3% (95% CI: 1.2%–5.4%). No cases of *P. ovale* were detected. HRP2-based RDT identified *P. vivax* as the predominant species, accounting for 15.7% (95% CI: 11.4%–20.0%), while *P. falciparum* constituted 3.3% (95% CI: 1.2%–5.4%). Similarly, microscopy detected *P. vivax* in 37 individuals (13.5%, 95% CI: 9.5%–17.6%), *P. falciparum* in 11 participants (4.0%, 95% CI, 1.7%–6.3%), and mixed infections (*P. falciparum* and *P. vivax*) in 7 participants (2.6%, 95% CI, 0.7%–4.4%) as shown in Fig. 3**.**

### 3.4 Performance of malaria prevalence HRP2-antigen based RDT compared with microscopy results

Among the 52 patients who tested positive using HRP2-antigen based RDT, three cases (two *P. falciparum* and one *P. vivax*) were confirmed negative by light microscopy. Conversely, out of 55 microscopy-confirmed positive cases, six were negative by HRP2-antigen based RDT (Table 3). The parasitemia levels among these microscopy-positive but HRP2-antigen based RDT -negative cases were ranged from 120 to 32,000 parasites/μL for *P. falciparum* and 160 to 18,000 parasites/μL for *P. vivax*.

**Table 3:** Diagnostic performance of HRP2-based RDTs against microscopy:Chi-square(χ^2^) test, kappa agreement, sensitivity, specificity, PPV, NPV, and overall accuracy.

RDT	Microscopy		χ2	Kappa	Sensitivity % [95%, CI]	Specificity % [95%, CI]	PPV % [95%, CI]	NPV % [95%,CI]	Accuracy %[95%,CI]
	Pos	Neg	*P* value	*P* value					
Pos	49	3	219.9	0.895	89.1(78.2–94.9)	98.6(96.0–99.6)	94.2(84.1–98.2)	97.3(94.2–98.8.4)	96.7(93.9–98.3)
Nege	6	216	0.00	0.00					

Pos: Positive, PPV: Positive predictive value, NPV: negative predictive value, Neg: Negative, CI: Confidence Intrval, χ2: chi-square value.

Using microscopy as the gold standard, the diagnosis of *Plasmodium* infection by HRP2-antigen based RDT demonstrated a sensitivity of 89.1% (95% CI: 78.2%– 94.9%) and a specificity 98.6% (95% CI: 97.0–100%). The corresponding PPV and NPV were 94.2% (95% CI: 87.8–100%) and 97.3% (95% CI: 95.2–99.4%), respectively and the overall test accuracy of 96.7% (93.9–98.3%) (χ2 = 219.9, *P* = 0.00). The kappa value of test agreement was 0.895 (89.5%) (Table 3).

### 3.5 Confirmation of *Plasmodium* species infection with PCR analysis

Discrepancies were observed between the diagnostics methods. Of the 64 cases detected by PCR, 13 were completely missed by the HRP2 antigen-based RDT and 9 cases were also missed by microscopy. On the other hand, three cases (two of *P. vivax* and one of *P. falciparum*) detected by the HRP2 antigen-based RDT were missed by microscopy, but the two *P. vivax* cases were confirmed positive by PCR.

**Fig. 4:**
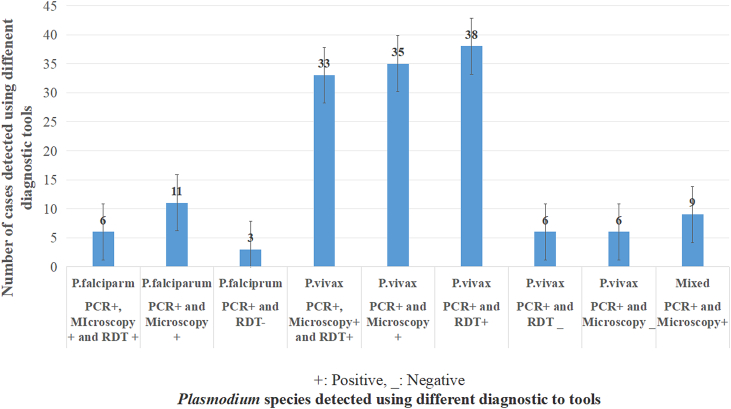
The discrepancies in *Plasmodium* species diagnosis across RDT, microscopy, and PCR.

Among the 11 *P. falciparum* cases identified via microscopy and PCR, four were not correctly detected by the HRP2-antigen based RDT. Similarly, all nine mixed-species infections confirmed by PCR were also identified as mixed by microscopy but were not detected by the HRP2-antigen-based RDT. The overall discrepancies in *Plasmodium* species-level diagnosis across HRP2-antigen-based RDT, microscopy, and PCR are summarized in Fig. 4.

The sensitivity (89.1%), and PPV (94.2%) were lower for HRP2-antigen based RDT compared to microscopy (Table 3). Similarly, this HRP2-antigen based RDT demonstrated lower sensitivity (79.7%) and NPV (94.1%) compared to PCR (χ2 = 200.2, *P* = 0.00). While, microscopy showed high specificity and PPV (100%), however, sensitivity was 85.9%, suggesting some true positives are missed compared to PCR. Overall accuracy was 96.7%, showing microscopy performs well compared to PCR (χ2 = 225.8, P = 0.00) (Table 4).

**Table 4:** Diagnostic performance of HRP2-based RDTs and microscopy against PCR:Chi-square(χ^2^) test, kappa agreement, sensitivity, specificity, PPV, NPV, and overall accuracy.

Diagnostic tools		PCR		χ2	kappa	Sensitivity %[95%, CI]	Specificity % [95%, CI]	PPV % [95%, CI]	NPV %[95%, CI]	Accuracy %[95%,CI]
		Pos	Neg	P value	P value					
RDT	Pos	51	1	200.2	0.847	79.7(68.3–87.7)	99.5(97.2–99.9)	98.1(89.9–99.9)	94.1 (90.2–96.5)	94.9(91.6–96.9)
	Neg	13	209	0.00	0.00					
Microscopy	Pos	55	0	225.8	0.904	85.9(75.4–92.4)	100(98.3–100)	100(93.5–100)	95.9(92.4–97.8)	96.7(93.9–98.3)
	Neg	9	210	0.00	0.00					

PPV: Positive predictive value, NPV: negative predictive value, CI: Confidence Intrval, PCR, polymerase chain reaction.

The level of agreement (Cohen's kappa) between the HRP2-antigen based RDT and PCR was 0.847 (84.7%). Similarly, the kappa value between the microscopy and PCR was 0.904 (90.4%) (P = 0.00) (Tables 4).

### 3.6 Correlation between hematological profile with malaria infection

A complete blood count, including total and differential WBC analysis, was performed for 274 patients clinically suspected of having malaria. The WBC counts among study participants ranged from 2000 to 27,000/μL, with a median value of 7600/μL (±4500). No significant correlation was observed between WBC count and malaria positivity (*r* = −0.08,*P* = 0.16). Similarly, RBC count showed no statistically significant association with malaria status (*r* = −0.1, *P* = 0.09).

In contrast, Hct and Hgb levels showed a weak yet statistically significant inverse relationship with malaria infection (*r* = −0.2, *P* = 0.001). Notably, platelet count showed a stronger negative correlation with malaria infection (*r* = −0.39, *P* < 0.001), suggesting that it may serve as a useful supportive hematological indicator of infection severity (Table 5).

**Table 5:** Correlation between hematological parameters and malaria infection status using PCR.

Parameter	Minimum	Maximum	Median (± SD	Pearson Correlation(r)	P-value
White Blood Cells (/μL)	2000	27,000	6400 (±4500)	**−0.08**	**0.160**
Granulocyte (%)	**8.50**	**94.00**	**71.35(±14.6)**	**0.08**	**0.207**
Lymphocyte(%)	**1.20**	**87.00**	**22.0 (±13.7)**	**−0.09**	**0.142**
Red Blood Cells (×10^6^/μL)	**1.30**	**14.50**	**4.80(±1.32)**	**−0.10**	**0.091**
Hematocrit(%)	**15.0**	**65.0**	**42.3(±7.37)**	**−0.20**	0.001*
Hemoglobin (g/dL)	**5.0**	**23.0**	**15.0(±2.59)**	**−0.20**	0.001*
Platelet Count (×10^3^/μL)	**5.60**	**926.0**	**194.50((±125.5)**	**−0.39**	0.00*

### 3.7 Bivariate analysis of associated factors of positive malaria results by PCR, microscopy, and RDT

Logistic regression analysis revealed that males were significantly more likely to be malaria-positive compared to females across all diagnostic methods. Specifically, males had 1.85 times higher odds of testing positive by RDT (OR = 1.85; 95% CI: 0.98–3.50), 1.9 times by PCR (OR = 1.90; 95% CI: 1.10–3.40), and 2.06 times by microscopy (OR = 2.06; 95% CI: 1.10–3.90).

Rural residence was also a significant predictor of malaria infection. Individuals residing in rural areas were 3.20 times more likely to test positive by PCR (OR = 3.20; 95% CI: 1.70–6.10), 3.89 times by microscopy (OR = 3.89; 95% CI: 1.90–7.90), and 5.50 times by RDT (OR = 5.50; 95% CI: 2.48–12.20) compared to their urban counterparts.

Family size greater than five was associated with significantly increased odds of malaria infection. Individuals from households with more than five members were 2.53 times more likely to be PCR-positive (OR = 2.53; 95% CI: 1.30–4.80) and 2.68 times more likely by microscopy (OR = 2.68; 95% CI: 1.40–5.20) compared to those from family size with fewer than five members.

Proximity to mosquito breeding sites was another key determinant. Individuals residing within 1000 m of a breeding site had higher odds of malaria infection: 2.52 times by PCR (OR = 2.52; 95% CI: 1.20–5.20), 2.59 times by microscopy (OR = 2.59; 95% CI: 1.19–5.69), and 3.43 times by RDT (OR = 3.43; 95% CI: 1.46–8.09), compared to those living more than 2000 m away.

Previous contact with malaria was significantly associated with increased infection risk. Individuals reporting prior malaria exposure had 4.74 times higher odds of being PCR-positive (OR = 4.74; 95% CI: 2.60–8.60), 6.83 times by RDT (OR = 6.83; 95% CI: 3.50–13.26), and 7.83 times by microscopy (OR = 7.83; 95% CI: 4.05–15.14) compared to those without such a history. Among these, individuals with relapse history had particularly elevated odds: 6.80 times by PCR (OR = 6.80; 95% CI: 2.30–20.10), 7.71 times by RDT (OR = 7.71; 95% CI: 2.40–24.76), and similar trends by microscopy.

Finally, febrile individuals were significantly more likely to test positive for *Plasmodium* spp. They had 4.74 times higher odds of testing positive by RDT (OR = 4.74; 95% CI: 2.45–9.18), 4.95 times by PCR (OR = 4.95; 95% CI: 2.70–9.10), and 5.33 times by microscopy (OR = 5.33; 95% CI: 2.77–10.28) compared to non-febrile individuals (Supplementary 3).

## 4 Discussion

Accurate and efficient diagnosis of malaria is crucial for early case detection, appropriate treatment, and interruption of disease transmission. However, this study revealed the challenges of accurately diagnosing malaria, as prevalence rates per diagnostic tool varied, affecting efforts aimed at elimination. A relatively higher malaria prevalence was detected by PCR at 23.4% (95% CI: 18.4%–28.4%), followed by microscopy at 20.1% (95% CI: 15.3%–24.8%), and HRP2-antigen-based RDT at 18.9.0% (95% CI: 14.3%–23.6%). These findings are consistent with previous studies conducted in Ethiopia (Tilahun et al., 2024; Golassa et al., 2013; Belachew et al., 2022a), and in other settings such as Zambia, Tanzania, and Papua New Guinea, where PCR has consistently outperformed microscopy and HRP2-antigen-based RDTs in detecting low-density and subpatent infections (Rosanas-Urgell et al., 2010; Sitali et al., 2019; Assefa et al., 2020; Belachew et al., 2022b; Mahende et al., 2016).

These discrepancies may be explained by differences in detection thresholds; for instance, PCR can detect as few as 0.1 parasites per milliliter of blood (Britton et al., 2016), whereas HRP2-antigen-based RDTs and microscopy needs higher parasitemia 50, and 100–200 parasites per milliliter, respectively (Tilahun et al., 2024; Kozycki et al., 2017a; Jimenez et al., 2017). However, in this study, the HRP2 antigen-based RDT failed to detect even higher parasite density (120 to 32,000 parasites/μL) for *P. falciparum* and (160 to 18,000 parasites/μL) for *P. vivax*. This finding suggests the possible presence of deletions in target genes, such as pfhrp2 (Kozycki et al., 2017a) and others like prozone effect.

In these findings the predominance of *P.vivax* across all diagnostic methods: 15.7% by HRP2-antigen-based RDTs, 13.5% by microscopy, and 16.1% by nPCR reflects a shifting epidemiological trend in Ethiopia. However, regard to species distribution, *Plasmodium falciparum* and *P. vivax* are the two dominant parasites in Ethiopia. National estimates suggest that *P. falciparum* typically predominates but that *P. vivax* also represents a substantial proportion of malaria cases, with its relative contribution varying across ecological zones and over time (Ketema et al., 2021; Assemie, 2022). This implies the broader epidemiological context helps situate the species patterns observed in our study, particularly where *P. vivax* appears relatively prominent within the study area compared to national averages. The relatively higher detection rate of *P.vivax* by HRP2 antigen-based RDT compared to the microscopy may be due to the presence of the antigen in the bloodstream after successful treatment, leading to false-positive results as supported by other studies (Ngasala et al., 2024; Bell et al., 2005; Kyabayinze et al., 2008; Tiono et al., 2014; Rogier et al., 2017). The predominance of observation of *P.vivax* aligns with findings from other studies in parts of Ethiopia (Ketema et al., 2021; Molla et al., 2024; Tadesse et al., 2018). However, studies in the Guba district and a systematic review indicated that *P. falciparum* had a significantly higher prevalence than *P.vivax* (Alkadir et al., 2020; Deress and Girma, 2019). This suggetes that *P. vivax* remains a significant concern due to its ability to cause relapses and complicate malaria elimination efforts. This highlights the varying prevalence across different regions and seasons.

Similarly*, P. falciparum* infection was detected in 3.2% (9/274) of participants using the HPR2 antigen-based RDT, 4% (11/274) using microscopy, and PCR. Among PCR-confirmed cases, 1.1% (3 of 274) of *P. falciparum* infections were incorrectly reported as negative by RDTs. The false negativity potentially due to low parasite density or the presence of specific genetic deletions in the malaria parasites of the test targets (Ikegbunam et al., 2024; Manjurano et al., 2021).

In this study, PCR revealed a higher prevalence of 3.3% (9/274) mixed infections, while microscopy identified 2.6% (7/274) mixed infections. These were confirmed as positive (mixed) by PCR; however, HRP2-antigen-based RDTs failed to detect these cases and only identified them as *P. vivax* (Fig. 3, Fig. 4). Another study also reported that microscopy remains the gold standard, identifying mixed infections that HRP2-antigen-based RDTs completely missed (Jimenez et al., 2017). A similar finding was observed in Ethiopia, where the HRP2-antigen-based RDT missed *P. falciparum* in 15 cases (Tilahun et al., 2024). Another study found that 5.4% of patients with confirmed *P. falciparum* infections by microscopy had negative RDT results (Koita et al., 2012). This higher *Plasmodium* detection discrepancy might be due to target gene deletions as supported by many reports (Golassa et al., 2020; Alemayehu et al., 2021; Kamaliddin et al., 2024). For instance, in some parts of Ethiopia, more than 57.8% of symptomatic patients had these deletions (Golassa et al., 2020; Alemayehu et al., 2021; Mekonen et al., 2024). According to a national survey in Ethiopia, approximately 22% of *P.falciparum* isolates showed complete deletions of the HPR2 and HPR3 genes (Kamaliddin et al., 2024). This gene deletion has reduced the sensitivity of HRP2-antigen based RDTs in detecting *P. falciparum* and mixed infections (Maltha et al., 2010; Nakavuma et al., 2023), significantly affecting diagnostic accuracy. The presence of these deletions poses a serious challenge to Ethiopia's malaria elimination goals by 2030, as undetected infections can lead to untreated cases (Golassa et al., 2020).

Generally, in this study 4.7% of false negative results were reported using HRP2-antigen-based RDTs, while 3.3% false negatives were reported using microscopy using PCR as a reference (Table 3, Table 4). Similar limitations due to their detection thresholds, which might miss submicroscopic infections (Oyegoke et al., 2022; Tilahun et al., 2024; Zimmerman and Howes, 2015). Others also reported that the sensitivity of HRP2-antigen based RDTs decreased from 88% to 67% as malaria transmission intensity fell (Kozycki et al., 2017a). The study showed that microscopy, though historically the gold standard, lacks sensitivity in detecting low-level parasitaemia (Matamoros et al., 2023; Martín-Díaz et al., 2018). This underscores the limitations of conventional methods and the need for context-specific diagnostic approaches. Additionally, other factors contributing to the reduced sensitivity of HRP2-antigen based RDTs include low antigen levels (low parasite densities) (Kozycki et al., 2017b), prozone effects, or gene deletions increasingly reported in East Africa (Feleke et al., 2022; Tafa et al., 2023; Maltha et al., 2012). Furthermore, it was also linked to low antigen persistence after treatment (Mouatcho and Goldring, 2013).

Despite these shortcomings, HRP2-antigen-based RDTs demonstrated a high positive predictive value (PPV) of 98.1% compared to the PCR reference, confirming their usefulness for positive malaria results. However, their lower sensitivity (89.1%) and limited accuracy (94.2%) showed caution in using them even for symptomatic patients (Table 4**)**. One study in Ethiopia reported an overall sensitivity of 67% for RDTs for diagnosing malaria compared to PCR (Belachew et al., 2022a), while another found sensitivities of 70%–77% for PfLDH-antigen based RDTs, despite relatively high specificity values (93% and 98%, respectively) (Alemayehu et al., 2020). In low-transmission settings like Eswatini (Swaziland), RDT sensitivity dropped to 51.7% and PPV to 67.3% (Ranadive et al., 2017). Similarly, in Indonesia, RDTs did not perform well in detecting low-density and non-falciparum infections (Unwin et al., 2020b). In contrast, in Senegal, the CareStart™ HRP2/pLDH Combo Test demonstrated high sensitivity (97.3%) and specificity (94.1%) in a low-transmission setting (Diallo et al., 2017b). A similar Indian study reported high overall performance of RDTs but noted reduced sensitivity in detecting mixed or non-falciparum infections, highlighting the need for continued refinement to improve their diagnostic applicability (Pande and Singh, 2021). Other studies highlight the limitation of RDT-based diagnostics (Stauffer et al., 2009; Gendrot et al., 2022; Wardhani et al., 2020; Fransisca et al., 2015; Houzé et al., 2013).

The present study also demonstrated lower agreement among diagnostic methods, with the highest concordance observed between PCR and microscopy (κ = 0.904), followed by PCR and RDT (κ = 0.847), and microscopy and HRP2-antigen based RDT (κ = 0.895) (Table 3, Table 4). These findings are consistent with results from other malaria-endemic regions (Awosolu et al., 2022; Bwire et al., 2019; Tegegn et al., 2024; Mawili-Mboumba et al., 2013; Nicastri et al., 2009). Similarly, a study in Ghana showed that the agreement between RDT and varATS qPCR was (κ = 0.571) compared to microscopy (κ = 0.409) (Opoku Afriyie et al., 2023). Even though microscopy has traditionally been the gold standard, its sensitivity is reduced in detecting low-parasitemia infections, an area where PCR is clearly superior (Mfuh et al., 2019). This finding indicates that the diagnostic sensitivity and specificity of the RDT fell below the WHO-recommended minimum threshold of 95% for *P.falciparum* diagnosis in endemic areas (WHO, 2021).

These findings collectively suggest that while microscopy and HPR2-antigen based RDTs are valuable for rapid, field-friendly diagnosis due to their high PPV, their limited sensitivity for diagnosing *Plasmodium* infections necessitates the development of more advanced diagnostics to support malaria control and elimination efforts in various transmission settings.

In this study, both WBC and RBC counts typically remained within normal ranges and showed no significant correlation with malaria positivity, consistent with findings from other regions (Muwonge et al., 2013; Lathia and Joshi, 2004). This highlights their limited usefulness as standalone diagnostic indicators. Alhough regional studies have reported correlations between specific hematological indices and malaria severity, such associations appear inconsistent and context-dependent (Antwi-Baffour et al., 2023; Seijas-Pereda et al., 2025).

Conversely, hematocrit and hemoglobin levels showed a weak but statistically significant negative correlation with malaria infection (*r* = −0.2, *P* = 0.001), suggesting mild anemia among infected individuals. This observation aligns with the known pathophysiological effects of malaria, particularly *P.falciparum* on red cell destruction and impaired erythropoiesis (Warrell et al., 2017).

Notably, platelet count showed a stronger negative correlation with *Plasmodium* infection status (*r* = −0.39, *P* < 0.001), reinforcing the role of thrombocytopenia as a more sensitive and consistent hematological marker. This finding is supported by previous studies from endemic areas that have identified a low platelet count as a useful adjunct in detecting malaria, particularly during acute infections (Lathia and Joshi, 2004; Chandra and Chandra, 2013). However, others reported that its reliability diminishes in uncomplicated cases, where significant differences in platelet counts may not be evident (Muwonge et al., 2013). This highlights the need for comprehensive diagnostic approaches that integrate clinical assessments and advanced testing methods.

Although these hematological changes are non-specific, they may provide supportive diagnostic value in clinical scenarios where parasitological confirmation is delayed or inconclusive. Among these, thrombocytopenia stands out as the most promising surrogate indicator of malaria infection in this population.

This study identifies several socio-demographic and environmental predictors that significantly influence malaria positivity across diagnostic methods including RDT, PCR, and microscopy. Consistent risk factors associated with increased odds of *Plasmodium* infection include being male, living in rural areas, having a larger household (family) size, being close to mosquito breeding sites, having a prior history of malaria, experiencing relapse episodes, and presenting with a fever were identified in this study.

Malaria positivity was consistently higher among males than females across all diagnostic methods. These findings are consistent with previous studies that have reported a higher incidence of malaria among males compared to females (Bhattacharya et al., 2013; Briggs et al., 2020). However, a community-based study has indicated a higher prevalence among females, suggesting that gender-specific exposure risks and social roles may vary across different epidemiological settings (Minwuyelet et al., 2024). The difference in gender malaria prevalence suggests that males may be at greater risk due to higher exposure, co-infection rates, and physiological factors that predispose them to more severe or persistent infections (Douglas et al., 2011). Biological sex-related differences may also contribute to varying susceptibility and immune responses to *Plasmodium* infections. For example, females have been shown to clear asymptomatic *P. falciparum* infections more effectively than males, potentially due to hormonal or immunological factors (Briggs et al., 2020). These gender-based differences highlight the importance of incorporating sex-specific analyses into malaria surveillance and intervention strategies to improve diagnostic accuracy and optimize control efforts.

Individuals in rural regions have a higher likelihood of testing positive for malaria using various diagnostic methods. Several findings support this study in other parts of Ethiopia (Golassa et al., 2013; Woldesenbet et al., 2024; Yutura et al., 2023; Tadesse et al., 2017). However, a significant proportion of malaria infections in urban settings are asymptomatic, with studies indicating that up to 96.4% of individuals with *P. falciparum* infections did not report recent fever (Sagna et al., 2023). Another study showed that urban agricultural practices create man-made habitats for malaria transmission even in urban settings. This indicates that both natural and artificial water sources are crucial in determining malaria distribution, whether in urban or more traditionally rural areas (Matthys et al., 2006). In Kinshasa, asymptomatic infections were significantly more prevalent in rural areas, suggesting urban settings may also harbor undetected reservoirs (Nundu et al., 2021). The difference in geographical distribution may be due to factors such as proximity to mosquito breeding sites, lack of preventive measures (e.g., insecticide-treated nets) and early treatment significantly contribute to higher infection rates in populations (Yutura et al., 2023; Tadesse et al., 2017).

Supporting the aforementioned idea, this study's findings reaffirmed that living near breeding sites like stagnant water elevates malaria risk, regardless of whether one is in urban or rural settings. Individuals residing within a kilometer of stagnant water had a higher likelihood of infection, further highlighting the significance of proximity in malaria transmission dynamics. (Alemu et al., 2011). Notably, results indicated that rural areas exhibited higher malaria positivity, underlining the influence of geographic and environmental factors on malaria transmission. Similarly, another study found that rural areas reported increased malaria positivity due to closer proximity to breeding sites such as stagnant water (Afolabi et al., 2023). In central-western Senegal, for example, rural areas with saline or hydromorphic soils, enabling water retention, have been identified as malaria hotspots. These conditions foster breeding sites for *Anopheles gambiae* mosquitoes, contributing to ongoing malaria transmission (Ndiaye et al., 2020).

This study explores the relationship between family size and malaria positivity, with findings indicating that larger families, particularly those with more than five members, indeed elevate the risk of malaria transmission. Similarly, other studies have reported that larger households may increase transmission risk, possibly due to greater exposure to mosquitoes and more feeding opportunities, thereby heightening the likelihood of malaria spread (Ojurongbe et al., 2023; Shcherbacheva and Haario, 2017; O'Meara et al., 2020). Socioeconomic factors, such as household wealth and education, can influence malaria risk. In certain instances, the relationship between household size and malaria risk is mediated by these factors, as observed in Mozambique, where household structure was linked to poverty (Searle et al., 2023). This supports the idea that household characteristics, such as size, could play a critical role in transmission dynamics, as they contribute to factors like the frequency of vector-human contact and potential exposure to infected mosquitoes.

Individuals with a history of prior malaria or relapse demonstrated significantly higher positivity rates. Similarly, a study reported that a history of previous malaria significantly increases the likelihood of current infection Additionally, studies showed that individuals with past malaria infections are at a greater risk of experiencing recurrences, often presenting as asymptomatic or submicroscopic cases, and a cohort study revealed that recurrences were detected significantly earlier using PCR compared to microscopy, highlighting the need for sensitive diagnostic tools (Garcia Castillo et al., 2024). In Western Kenya, qPCR) revealed a higher rate of parasite positivity compared to microscopy, especially in submicroscopic infections, underscoring the need for sensitive diagnostic methods to detect low parasitemia (Lo et al., 2015). A study involving controlled human malaria infection showed that individuals with more previous exposure to malaria had better control over parasitemia, indicating that past infections might influence current immune responses (Achan et al., 2020).

A case study highlighted the potential for *P. falciparum* to persist for years after initial infection, suggesting that low-grade parasitemia can go unnoticed and later cause recrudescence or cryptic malaria (Marino et al., 2023). This phenomenon is significant in understanding malaria transmission and control, as these low-level infections can serve as reservoirs for the disease, contributing to ongoing transmission cycles. The persistence of such infections is influenced by various factors, including the type of malaria parasite and the diagnostic methods used.

The strong association of febrile status across all diagnostic methods, despite the variability in odds ratios, highlights the inherent challenges and discrepancies in diagnostic sensitivity observed in clinical research. Individuals presenting with fever have greater odds of testing positive for malaria (Deress and Girma, 2019). For instance, HRP2-based RDTs, though operationally convenient, can produce false positives due to persistent antigenemia or false negatives due to pfhrp2 gene deletions, an emerging threat in malaria-endemic areas (Martiáñez-Vendrell et al., 2022; Apinjoh et al., 2024; Pasquier et al., 2020). On the other hand, PCR, while more sensitive, is limited by cost, infrastructure, and turnaround time, hindering its routine use in low-resource settings. The consistency of risk factors across diagnostic methods reinforces the validity of these associations but also underscores the continuing diagnostic challenges, especially in detecting asymptomatic or low-density infections.

## 5 Conclusions and recommendations

This study highlights a significant burden of malaria among clinically suspected patients in Bichena primary hospital northwest Ethiopia, with *P. vivax* emerging as the dominant species across all diagnostic methods. The prevalence of *Plasmodium* infection ranged from 18.90% as detected by RDT to 23.4% by PCR, discrepancies between diagnostic methods were evident. RDTs failed to detect *P. falciparum* and mixed-species infections identified by microscopy and PCR. Even though microscopy showed better agreement with PCR but also missed a small number of cases detected by PCR analysis. These findings emphasize the limitations of relying on a single diagnostic tool in endemic settings, especially where *P. falciparum* and mixed infections are common. Hematological markers such as platelet count and hemoglobin levels showed inverse associations with infection, suggesting their potential value as adjunctive indicators.

To address diagnostic gaps, it is recommended to integrate more sensitive molecular methods into surveillance and confirmatory testing, particularly for submicroscopic or mixed-species infections. Strengthening microscopy training and quality assurance is also crucial for improving detection accuracy. Expanding ITN coverage, enhancing community awareness, and utilizing hematological indicators for clinical suspicion can further enhance early detection and case management.

## Consent to publications

All the authors have read the final manuscript and provided consent for publication.

## CRediT authorship contribution statement

**Awoke Minwuyelet:** Writing – review & editing, Writing – original draft, Visualization, Validation, Supervision, Software, Resources, Project administration, Methodology, Investigation, Formal analysis, Data curation, Conceptualization. **Delenasaw Yewhalaw:** Writing – review & editing, Supervision, Investigation, Conceptualization. **Getnet Atenafu:** Writing – review & editing, Supervision, Conceptualization.

## Ethics approval and consent to participate

Ethical approval was obtained from the Institutional Review Board (IRB) of Debre Markos University, Research and Technology Transfer Directorate, on 30 December 2024 (Ref. No:DMU/RTTD/75/10/24; Protocol No: DMU/002/2017). Verbal informed consent was obtained from all participants prior to enrollment, in accordance with the approved ethical guideline.

## Funding

No funding

## Declaration of competing interest

The authors declare no conflicts of interest.

## Data Availability

All the data generated during this study are included in this manuscript and supplementary files.

## Appendix A Supplementary data

Supplementary material 1: **Protocols for Assessing Diagnostic Challenges in Malaria detection : using Nested PCR Analysis of *Plasmodium* Parasites from DBS samples** Image 1

Supplementary material 2: Ethical aproval letter Image 2

Supplementary material 3: Univariate analysis of predictors of positive malaria results by PCR, blood smear.and RDT Image 3
