# Supplementary material for: Diagnostic challenges in malaria detection: A comparative diagnostic performance of HRP2-based rapid diagnostic tests, microscopy, and PCR at Bichena primary hospital, Northwest Ethiopia
Source: Parasite Epidemiol Control. 2026 Feb 19;33:e00485. doi: 10.1016/j.parepi.2026.e00485 (PMC12950475; doi:10.1016/j.parepi.2026.e00485)
Supplement: Supplementary file 1 — Supplementary material 1: Protocols for Assessing Diagnostic Challenges in Malaria detection : using Nested PCR Analysis of Plasmodium Parasites from DBS samples [file mmc1.docx]

**Supplementary file 1: Protocols for Assessing Diagnostic Challenges in Malaria: using Nested PCR Analysis of *Plasmodium* Parasites from DBS samples**

**Annex 1. DNA extraction procedure using Chelex-100 isolation of DNA from DBS samples**

**DNA extraction procedure**

Malaria diagnosis was also conducted using nPCR. For molecular analysis, genomic DNA was extracted using the Chelex-100 method, as previously described by Wooden et al.[1], with a few slight modifications. The 3 mm DBS sample was punched out and placed into pre-labeled 1.5 mL Eppendorf tubes separately. Subsequently, 50 μL of 10% saponin solution and 950 μL of phosphate-buffered saline (PBS) were added to each tube containing a DBS sample. The tubes were then capped, vortexed briefly, and incubated overnight at 4 °C.

After incubation, the samples were centrifuged at 14,000 rpm for 10 minutes, and the supernatant was carefully discarded. To remove residual saponin, 1,000 μL of PBS was added to each tube and centrifuged at 14,000 rpm for 5 minutes. The supernatant was again discarded by pipetting, and the pellet was allowed to air-dry at room temperature for 15 minutes. Subsequently, 150 μL of 20% Chelex® resin solution and 100 μL of distilled water were added to the dried pellet.

Finally, the parasite DNA was extracted by incubating the mixture in 95 °C for 10 minutes in a heat block, with intermittent vortexing every 2 minutes throughout the incubation period. The incubated DNA mixture was centrifuged at 14,000 rpm for 1 minute to pellet the Chelex resin, resulting in the release of parasite DNA into the supernatant. A total of 100 μL of the extracted DNA was carefully transferred into a new labeled 1.5 mL Eppendorf tube and stored at −20 °C until the nPCR assay was performed.

**DNA Amplification procedure**

The 18S rRNA gene of the *Plasmodium* genus was amplified using a TC9639 thermal cycler (Benchmark Scientific, Sayreville, NJ, USA) [2]. To achieve this, the ToughMix® was used and it is a ready-to-use master mix containing optimized concentrations of MgCl₂, dNTPs (dATP, dCTP, dGTP, dTTP), hot-start DNA polymerase, AccuVue™ blue qPCR dye, and stabilizers [3].

Genus-level amplification was conducted using a 25 μL reaction mixture consisting of 2 μL of extracted template DNA, 0.4 μM each of the forward (rPLU5) and reverse (rPLU6) primers, 6 μL of PerfeCTa® qPCR ToughMix® Low ROX™ (Quantabio), and 16.2 μL of nuclease-free water ( **Table 1**).

**Table 1: Nested PCR Mix formulation for first round Nested PCR amplification (N1) of the 18S ribosomal RNA gene (for genus level detection).**

| No. | nPCR Ingredients | Volume/Reaction (ul) (N1) | Volume/26 Reactions (ul)  (N1) | Remark |
| --- | --- | --- | --- | --- |
| 1. | Nuclease free water | 16.2 | 421.2 |  |
| 2. | Primer (Forward) | 0.4 (rPLU5) | 10.4 |  |
| 3. | Primer (Reverse) | 0.4 (rPLU6) | 10.4 |  |
| 4. | PerfeCTa® qPCR ToughMix® Low ROX™ (Quantabio) | 6 | 156 |  |
|  | Aliquot (PCR Mix) |  | 23 |  |
|  | DNA Template | 2 | 2 |  |
|  | Total volume |  | 25 |  |

After carefully preparing the PCR mixture, it was inserted into the TC 9639 thermal cycler. The thermal cycling conditions were set to an initial denaturation at 95°C for 10 minutes, followed by 35 cycles consisting of denaturation at 95°C for 60 seconds, annealing at 58°C for 60 seconds, and extension at 72°C for 90 seconds. The process included a final extension at 72°C for 10 minutes, with a holding period of over 4 hours at 10°C. Each PCR run incorporated a negative control in which 1 µL of PCR-grade water replaced the template DNA to check for contamination [4] ( **Table 2).**

**Table 2: Nested PCR amplification program for both rounds of amplifications (N1 and N2)**

|  | nPCR Program (N1 and N2) | | | |  |
| --- | --- | --- | --- | --- | --- |
| Step | Cycle | Temp. (^o^C) | Period | Purpose | No. of Cycles |
| 1^st^ | 1 | 95 | 10min | Initial Denaturation | 1 |
| 2^nd^ | 1 | 95 | 60sec | Denaturation | x30 for N1 and N2 |
|  | 2 | 58 | 60sec | Annealing |  |
|  | 3 | 72 | 90sec | Extension |  |
| 3^rd^ | 1 | 72 | 10min | Final extension | 1 |
| 4^th^ |  | 10 | >4hrs | Hold N1/N2 product |  |

After genus-level detection, a second round of amplification was carried out using species-specific primers designed to simultaneously detect and differentiate *P. falciparum, P. vivax,* and *P. ovale* [5, 6]. Notably, template DNA was used in this step to minimize non-specific amplification and reduce the risk of primer-dimer formation when N1 amplicon was used. The primers and their sequence were used according to Snounou et al [7] (**Table 3**).

**Table 3. Genus and species-specific primers for confirmation of *P. falciparum, P.vivax andP.ovale*) infection by Nested PCR amplification of the 18S ribosomal RNA gene**

| Name of species | Sequences (5′-3′) | Band size | References |
| --- | --- | --- | --- |
| rPLU5 | CCT GTT GTT GCC TTA AAC TTC | 1.5-1.6kb  (1.2kbp) | Snounou, G. et al. 2002 [7] |
| rPLU6 | TTA AAA TTG TTG CAG TTA AAA CG |  |  |
| *P. falciparum* : rFAL1 | TTA AAC TGG TTT GGG AAA ACC AAA TAT ATT | 206bp |  |
| *P.falciparum*: rFAL2 | ACA CAA TGA ACT CAA TCA TGA CTA CCC GTC |  |  |
| *P.vivax*::rVIV1 | CGC TTC TAG CTT AAT CCA CAT AAC TGA TAC | 121bp |  |
| *P.vivax*: rVIV2 | ACT TCC AAG CCG AAG CAA AGA AAG TCC TTA |  |  |
| *P.ovale*:rOVA1 | ATC TCT TTT GCT ATT TTT TAG  TAT TGG AGA | 780-800bp |  |
| *P oval*:rOVA2(rPLU2) | ATC TAA GAA TTT CAC CTC TGA CAT CTG |  |  |

Similar with the genus level, nPCR amplification was performed in a total reaction volume of 25 µL using a 96-well nPCR plate. However, the reaction mixture contained 6 µL of PerfeCTa® qPCR ToughMix® Low ROX™ (Quantabio) master mix, 0.4 µL of each species-specific forward and reverse primer pair rFAL1/rFAL2 (*P.falciparum*), rVIV1/rVIV2 *(P.vivax*), and rOVA1/rOVA2 for *P. ovale,* 2 µL of template DNA, and 14.6 µL of nuclease-free water (**Table 4**).

**Table 4: Nested PCR Mix formulation for second round Nested PCR amplification (N2) of the 18S ribosomal RNA gene.**

| No. | nPCR Ingredients | Volume/Rxn (ul) (N2) | Volume/ 26 Rxns (ul) (N2) | Remark |
| --- | --- | --- | --- | --- |
| 1. | Nuclease free water | 14.6 | 379.6 |  |
| 2. | Primer (Forward) | 0.4 (rFAL1) | 10.4 |  |
|  | Primer (Reverse) | 0.4(rFAL2) | 10.4 |  |
| 3 | Primer (Forward) | 0.4(rVIV1) | 10.4 |  |
|  | Primer (Reverse) | 0.4(rVIV2) | 10.4 |  |
| 4 | Primer (Forward) | 0.4(rOVA1) | 10.4 |  |
|  | Primer (Reverse) | 0.4(rOVA2) | 10.4 |  |
| 5. | PerfeCTa® qPCR ToughMix® Low ROX™ (Quantabio) | 6 | 156 |  |
|  | Aliquot (PCR Mix) |  | 23 |  |
|  | DNA Template | 2 | 2 |  |
|  | Total volume |  | 25 |  |

The thermal cycling condition was similar to that at the genus level, except it was adjusted for 30 cycles. The total runtime for the reaction was approximately 2 hours and 37 minutes.

Annex 3. **Visualization of Amplified DNA on a 1.5% Agarose Ge**

A 1.5% (w/v) agarose gel was prepared by dissolving 1.5 g of agarose (VWR®) in 100 mL of 1× TAE buffer (VWR® UltraPure), heating it in a microwave for ~2 minutes. After the solution cooled down, 1 µL of Ethidium bromide (Invitrogen™ UltraPure™) was added and mixed. The gel was poured into a casting tray with combs of two well sizes and allowed to solidify at room temperature (~20 min). Finally, combs were removed, and the gel (in tray) was placed in an electrophoresis chamber filled with 1× TAE buffer until fully submerged.

After preparing the gel, 10 µL of PCR 100 bp Low Ladder (SIGMA) was loaded into 2-3 lanes as molecular size markers. The remaining lanes were loaded by 10 µL of amplified PCR products. The gel tray was properly connected to the electrophoresis unit, and electrophoresis was run at 90 V and 400 mA for 75 minutes. Finally, DNA bands were visualized using a UV transilluminator (PhotoDoc-It™ Imaging System), and *Plasmodium* species were identified by comparing band sizes to the DNA ladder (**Species level,** Figure 1).


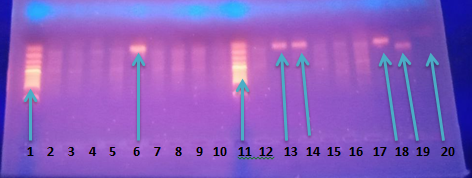


**Figure 1. Nested PCR gel-electrophoresis *Plasmodium* species amplification result.**

This gel electrophoresis image showed nPCR amplification products for the detection of *Plasmodium vivax* and *Plasmodium falciparum* using species-specific primers. Lanes 1 and 11 (M): 100 bp DNA ladder used as a molecular size reference. Lanes 6, 13, 14, and 18: Positive bands at approximately 121 bp, indicating the presence of *P. vivax* DNA. Lane 19: Positive control for *P. falciparum*, showing a distinct band at ~205 bp. Lanes 2–5, 7–10, 12, 15–17: No visible bands, indicating negative nPCR results or undetectable parasite DNA. Lane 20: Negative control showing no amplification, confirming absence of contamination

**References**

1. Wooden J, Kyes S, Sibley C. PCR and strain identification in Plasmodium falciparum. Parasitology today. 1993;9(8):303-5.

2. Chua KH, Lee PC, Chai HC. Development of insulated isothermal PCR for rapid on-site malaria detection. Malaria Journal. 2016;15(1):134.

3. Kamau E, Ockenhouse CF, Feghali KC, Alemayehu S. Sensitive Multiplex QPCR Assay For The Detection of Malaria. Google Patents; 2015.

4. Bharti AR, Patra KP, Chuquiyauri R, Kosek M, Gilman RH, Llanos-Cuentas A, et al. Polymerase chain reaction detection of Plasmodium vivax and Plasmodium falciparum DNA from stored serum samples: implications for retrospective diagnosis of malaria. American Journal of Tropical Medicine and Hygiene. 2007;77(3):444-6.

5. Rosanas-Urgell A, Mueller D, Betuela I, Barnadas C, Iga J, Zimmerman PA, et al. Comparison of diagnostic methods for the detection and quantification of the four sympatric Plasmodium species in field samples from Papua New Guinea. Malaria Journal. 2010;9:1-8.

6. Veron V, Simon S, Carme B. Multiplex real-time PCR detection of P. falciparum, P. vivax and P. malariae in human blood samples. Experimental parasitology. 2009;121(4):346-51.

7. Snounou G, Singh B. Nested PCR analysis of Plasmodium parasites. Malaria Methods and Protocols: Methods and Protocols: Springer; 2002. p. 189-203.
