# Supplementary material for: Diagnostic challenges in malaria detection: A comparative diagnostic performance of HRP2-based rapid diagnostic tests, microscopy, and PCR at Bichena primary hospital, Northwest Ethiopia
Source: Parasite Epidemiol Control. 2026 Feb 19;33:e00485. doi: 10.1016/j.parepi.2026.e00485 (PMC12950475; doi:10.1016/j.parepi.2026.e00485)
Supplement: Supplementary file 2 — Supplementary material 2: Ethical aproval letter [file mmc2.pdf]

በኢትዮጵያ ፌደራላዊ ዲሞክራሲያዊ ሪፐብሊክ
ትምህርት ሚኒስቴር
የደብረ ማርቆስ ዩኒቨርሲቲ
የምርምርና ቴክኖሎጂ ሽግግር ዳይሬክቶሬት

The Federal Democratic Republic Of Ethiopi
Ministry of Education
DebreMarkos University
Research & Technology Transfer Directora

Ref No: DMU /RTTD/[illegible]/10/24

Date: 30 December 2024

To: Mr. Awoke Minwuyelet (PI)

Debre Markos University

Subject: Ethical Clearance

Your research proposal entitled **"Epidemiology, Diagnostic Challenges of Malariaand the Effect of Artemether-Lumefantrine with a Single Dose of Primaquine Treatment on Anopheles Mosquito Infectivity Among Malaria Suspected Patients Attending Bichena Primary Hospital, Amhara Region, Ethiopia"** with protocol number **DMU/002/2017** has been reviewed by the Institutional Research Ethics Review Committee of Debre Markos University for its ethical soundness and it is found to be ethically acceptable.

Thus, based on the recommendation of the Institutional Research Ethics Review Committee, the Research and Technology Transfer Directorate has awarded this Ethical Clearance for the above-stated study to be reported by **Mr. Awoke Minwuyelet** as principal investigator and **Dr. Getnet Atenafu, Professor DelenasawYehalaw and Professor Andrea Sciarretta** as co-investigators as of 27 December 2024. The Ethical clearance is valid for **one year**; if deemed necessary, the researcher should ask to renew the clearance.

The investigator must submit his progress report to the Research and Technology Transfer Directorate of Debre Markos University every 12 months from the date of initial/renewal ethical approval and during the study as required.

Debre Markos University Research & Technology Transfer Directorate

Sincerely yours!

የምርምርና ቴክኖሎጂ ሽግግር ዳይሬክቶሬት ዳይሬክተር
Director for Research & Technology Transfer Directorate

C.C.

Institutional Research Ethics Review Committee (IRERC)
Debre Markos University

☎ +251 (0) 58-771-6002/ 058-778-0809 Fax: +251 (0) 58-771-1764 ✉ 269

መልስ ሲጽፉልን እባክዎን የእኛን ቁጥር ይጥቀሱ
In replying Please Quote our Ref.No.
