# Supplementary material for: Diagnostic challenges in malaria detection: A comparative diagnostic performance of HRP2-based rapid diagnostic tests, microscopy, and PCR at Bichena primary hospital, Northwest Ethiopia
Source: Parasite Epidemiol Control. 2026 Feb 19;33:e00485. doi: 10.1016/j.parepi.2026.e00485 (PMC12950475; doi:10.1016/j.parepi.2026.e00485)
Supplement: Supplementary file 3 — Supplementary material 3: Univariate analysis of predictors of positive malaria results by PCR, blood smear.and RDT [file mmc3.docx]

Supplementary file 3: Univariate analysis of predictors of positive malaria results by PCR, blood smear.and RDT

| Characteristic | | PCR Pos (%) | OR (95% CI) | P-value | Microscopy pos (%) | OR (95% CI) | P-value | RDT pos (%) | OR (95% CI) | P-value |
| --- | --- | --- | --- | --- | --- | --- | --- | --- | --- | --- |
| sex | Male | 43(28.3) | 1.9(1.1-3.4) | 0.03* | 38(25) | 2.06(1.1-3.9) | 0.025* | 35(23.0) | 1.85(0.98-3.5) | 0.059 |
|  | Female | 21(17.2) | 1 |  | 17(13.9) | 1 |  | 17(13.9) |  |  |
| Age categories | <5 | 11(36.7) | 2.03(0.9-4.6) | 0.08 | 9(30) | 1.83(0.8-4.3) | 0.16 | 7(23.3) | 1.34(0.54-3.3) | 0.53 |
|  | 5-14 | 4(17.4) | 0.7(0.2-2.3) | 0.59 | 4(17.4) | 0.89(0.3-2.8) | 0.85 | 4(17.4) | 0.92(0.29-2.86) | 0.89 |
|  | >15 | 49(22.2) | 1 |  | 42(19.0) | 1 |  | 41(18.6) | 1 |  |
| Residence | Rural | 50(31.6) | 3.20(1.7-6.1) | 0.00* | 44(28.4) | 3.89(1.9-7.9) | 0.00* | 44(28.4) | 5.5(2.48-12.2) | 0.00* |
|  | urban | 14(12.6) | 1 |  | 11(9.2) | 1 |  | 8(6.7) | 1 |  |
| Marital status | single | 14(25.9) | 1.67(0.42-6.71) | 0. .47 | 12(22.2) | 1.37(0.34-5.56) | 0.66 | 13(24.1) | 1.52(0.38-6.12) | 0. 56 |
|  | married | 33(21.3) | (1.27(0.34-4.67) | 0.72 | 27(18) | 1.02(0.27-3.81) | 0.97 | 25(16.7) | 0.93(0.25-3.49) | 0. 92 |
|  | Windowed/divorced | 4(22.2) | 1 |  | 3(17.6) | 1 |  | 3(17.6) | 1 |  |
| Education status | Illiterate | 23(23.5) | 0.89(0.4-1.9) | 0.78 | 21(21.4) | 1.23(0.5-2.8) | 0.63 | 18(18.4) | 0.90(0.39-2.08) | 0.81 |
|  | Read and write | 12(19.7) | 0.72(0.3-1.7) | 0.46 | 11(18.0) | 0.99(0.4-2.6) | 0.98 | 9(14.8) | 0.69(0.26-1.82) | 0.457 |
|  | Up to high school | 15(25) | 0.98(0.4-2.3) | 0.95 | 13(21.7) | 1.24(0.5-3.1) | 0.64 | 14(23.3) | 1.22(0.49-2.97) | 0.66 |
|  | Graduated | 14(27.3) | 1 |  | 10(18.2) | 1 |  | 11(20) | 1 |  |
| Household level | >5 | 21(38.2) | 2.53(1.34-4.75) | 0.00* | 19(34.5) | 2.68(1.4-5.2) | 0.003* | 19(34.5) | 2.97(1.53-5.8) | 0.001* |
|  | <5 | 42(19.6) | 1 |  | 36(16.4) | 1 |  | 33(15.1) | 1 |  |
| Malaria prevention method | ITNs | 22(22.10 | 0.89(0.5-1.6) | 0.72 | 117(17.8) | 0.81(0.4-1.5) | 0.51 | 15(15.8) | 0.72(0.37-1.39) | 0.33 |
|  | Not use any thing | 43 (24.0) | 1 |  | 38(21.2) | 1 |  | 37(20.7) | 1 |  |
| Current use of ITNs | Daily | 19(21.8) | 0.47(0.1-2.13) | 0.32 | 17(19.5) | 1.21(0.13-11.08) | 0.86 | 15(17.2) | 1.04(0.11-9.57) | 0.97 |
|  | sometimes | 3(50) | 1 |  | 1(16.7) | 1 |  | 1(16.7) | 1 |  |
| Mosquito Breeding sites | <1000m | 44(28.3) | 2.52(1.2-5.2) | 0.012* | 38.8(24.5) | 2.59(1.19-5.69) | 0.017* | 38(24.50 | 3.43(1.46-8.09) | 0.005* |
|  | 1000-2000m | 9(23.7) | 1.97(0.7-5.3) | 0.17 | 8(21.1) | 2.13(0.75-6.05) | 0.155 | 17(18.4) | 2.39(0.77-7.39) | 0.13 |
|  | >2000m | 11(13.6) | 1 |  | 9(11.1) | 1 |  | 7(8.6) | 1 |  |
| Ever malaria contacted | Yes | 39(42.8) | 4.74(2.6-8.6) | 0.00* | 39(42.8) | 7.83(4.05-15.14) | 0.00* | 36(39.6) | 6.83(3.5-13.26) | 0.00* |
|  | No | 25(13.7) | 1 |  | 16(8.7) | 1 |  | 16(8.7) | 1 |  |
| Relapse History of malaria | Yes | 34(56.7) | 6.8(2.3-20.1) | 0.001* | 34(56.7) | 6.8(2.3-20.1) | 0.001* | 32(53.3) | 7.71(2.4-24.76) | 0.001* |
|  | No | 5(16.1) | 1 |  | 5(16.1) | 1 |  | 4(12.9) | 1 |  |
| Body Temperature | Febrile | 45(39.8) | 4.95(2.7-9.1) | 0.00* | 40(35.4) | 5.33(2.77-10.28) | 0.00* | 37(32.7) | 4.74(2.45-9.18) | 0.00* |
|  | Non-febrile | 19(11.8) | 1 |  | 15(9.3) | 1 |  | 15(9.1) | 1 |  |

OR: Odd ratio, PCR: Polymerase chain reaction, Pos: Positive, RDT: Rapid diagnosis test, CI: confidence Interval
